# Heterogeneity of ILC2s in the Intestine; Homeostasis and Pathology

**DOI:** 10.3389/fimmu.2022.867351

**Published:** 2022-05-30

**Authors:** Shogo Sunaga, Junya Tsunoda, Toshiaki Teratani, Yohei Mikami, Takanori Kanai

**Affiliations:** ^1^Division of Gastroenterology and Hepatology, Department of Internal Medicine, Keio University School of Medicine, Shinjuku-ku, Tokyo, Japan; ^2^Department of Surgery, Keio University School of Medicine, Tokyo, Japan; ^3^AMED-CREST, Japan Agency for Medical Research and Development, Tokyo, Japan

**Keywords:** ILC2 - group 2 innate lymphoid cell, IBD - inflammatory bowel disease, Crohn’s disease, ulcerative colitis, mucosal immunology

## Abstract

Group 2 innate lymphoid cells (ILC2s) were identified in 2010 as a novel lymphocyte subset lacking antigen receptors, such as T-cell or B-cell receptors. ILC2s induce local immune responses characterized by producing type 2 cytokines and play essential roles for maintaining tissue homeostasis. ILC2s are distributed across various organs, including the intestine where immune cells are continuously exposed to external antigens. Followed by luminal antigen stimulation, intestinal epithelial cells produce alarmins, such as IL-25, IL-33, and thymic stromal lymphopoietin, and activate ILC2s to expand and produce cytokines. In the context of parasite infection, the tuft cell lining in the epithelium has been revealed as a dominant source of intestinal IL-25 and possesses the capability to regulate ILC2 homeostasis. Neuronal systems also regulate ILC2s through neuropeptides and neurotransmitters, and interact with ILC2s bidirectionally, a process termed “neuro-immune crosstalk”. Activated ILC2s produce type 2 cytokines, which contribute to epithelial barrier function, clearance of luminal antigens and tissue repair, while ILC2s are also involved in chronic inflammation and tissue fibrosis. Recent studies have shed light on the contribution of ILC2s to inflammatory bowel diseases, mainly comprising ulcerative colitis and Crohn’s disease, as defined by chronic immune activation and inflammation. Modern single-cell analysis techniques provide a tissue-specific picture of ILC2s and their roles in regulating homeostasis in each organ. Particularly, single-cell analysis helps our understanding of the uniqueness and commonness of ILC2s across tissues and opens the novel research area of ILC2 heterogeneity. ILC2s are classified into different phenotypes depending on tissue and phase of inflammation, mainly inflammatory and natural ILC2 cells. ILC2s can also switch phenotype to ILC1- or ILC3-like subsets. Hence, recent studies have revealed the heterogeneity and plasticity of ILC2, which indicate dynamicity of inflammation and the immune system. In this review, we describe the regulatory mechanisms, function, and pathological roles of ILC2s in the intestine.

## Introduction

The intestine is one of the largest organs continually exposed to the external environment and it harbors an immune system to protect the host from pathobionts (1). Innate lymphoid cells (ILCs) are newly classified lymphocyte subsets that serve as a frontline defense, particularly in the mucosal tissues (2, 3). Unlike T- and B-cells, ILCs do not express adaptive antigen recognition receptors, and as such their expansion and activation are not driven in an antigen-specific manner, but rather by cytokine signals in the local microenvironment in each tissue. Although ILCs cannot induce antigen-specific reactions, they quickly respond to external antigen from the local microenvironment and rapidly produce various cytokines including interleukins (IL) and interferon (IFN) to maintain tissue homeostasis. ILCs are classified into three groups based on lineage-determining transcription factors and cytokine production, mirroring T helper cell subsets (2, 3). Group 2 ILCs (ILC2s) require transcription factors GATA3 (4, 5) and RORα (6, 7) for differentiation and produce signature “Type 2” cytokines, such as IL-4, IL-5, IL-9, and IL-13, as well as IL-6, IL-10, IL-17, and amphiregulin (AREG) (8–12). ILC2s were first reported as natural helper cells, nuocytes, and innate type 2 helper cells, and were detected in mesenteric adipose tissue, mesenteric lymph nodes, spleen, liver, lung, and small intestine (13–15). More recently, ILC2s have been found in various organs that are confronted with external antigen, such as the intestine, respiratory system, and skin, and also in those that are not continually challenged, such as liver, heart, muscle, and brain (16). Recent advancement of single cell omics and mass cytometry technologies have revealed that ILC2s possess tissue-specific phenotypes and contribute to the tissue-specific regulation of inflammation, allergic immunity, parasite infection, metabolism, and tissue repair (16–19).

In the intestine, epithelial cells respond to bacteria, parasites, and allergen within the intestinal lumen, and produce alarmins, such as IL-25, IL-33, and thymic stromal lymphopoietin (TSLP), which subsequently activate ILC2s to proliferate and produce cytokines (16). Recent studies have reported abundant regulation of ILC2s beyond alarmins, including neuro-immune crosstalk, which is mediated by neurotransmitters and cytokines. Activation of tissue-resident ILC2s causes not only local inflammation but also subsequent tissue remodeling and organ fibrosis associated with intestinal chronic inflammatory conditions, such as inflammatory bowel disease (IBD), including ulcerative colitis (UC) or Crohn’s disease (CD) (17). However, negative clinical trial results regarding targeting type 2 immune responses have encouraged us to explore the complexity of ILC2 and other type 2 immune cells, and their cytokine production. New technologies, including single-cell analysis, have been used to better decipher the functions and heterogeneity of ILC2s. Initially, ILC2s were thought to have roles in defending against parasitic infection and promoting allergic pathology (20), whereas studies of ILC2s in the context of IBD are developing. In this review, we focus on the roles of ILC2s in the intestine and discuss their regulation, neuroimmunology, fibrosis, and contribution to IBD.

## Regulation of ILC2

**Figure 1:**
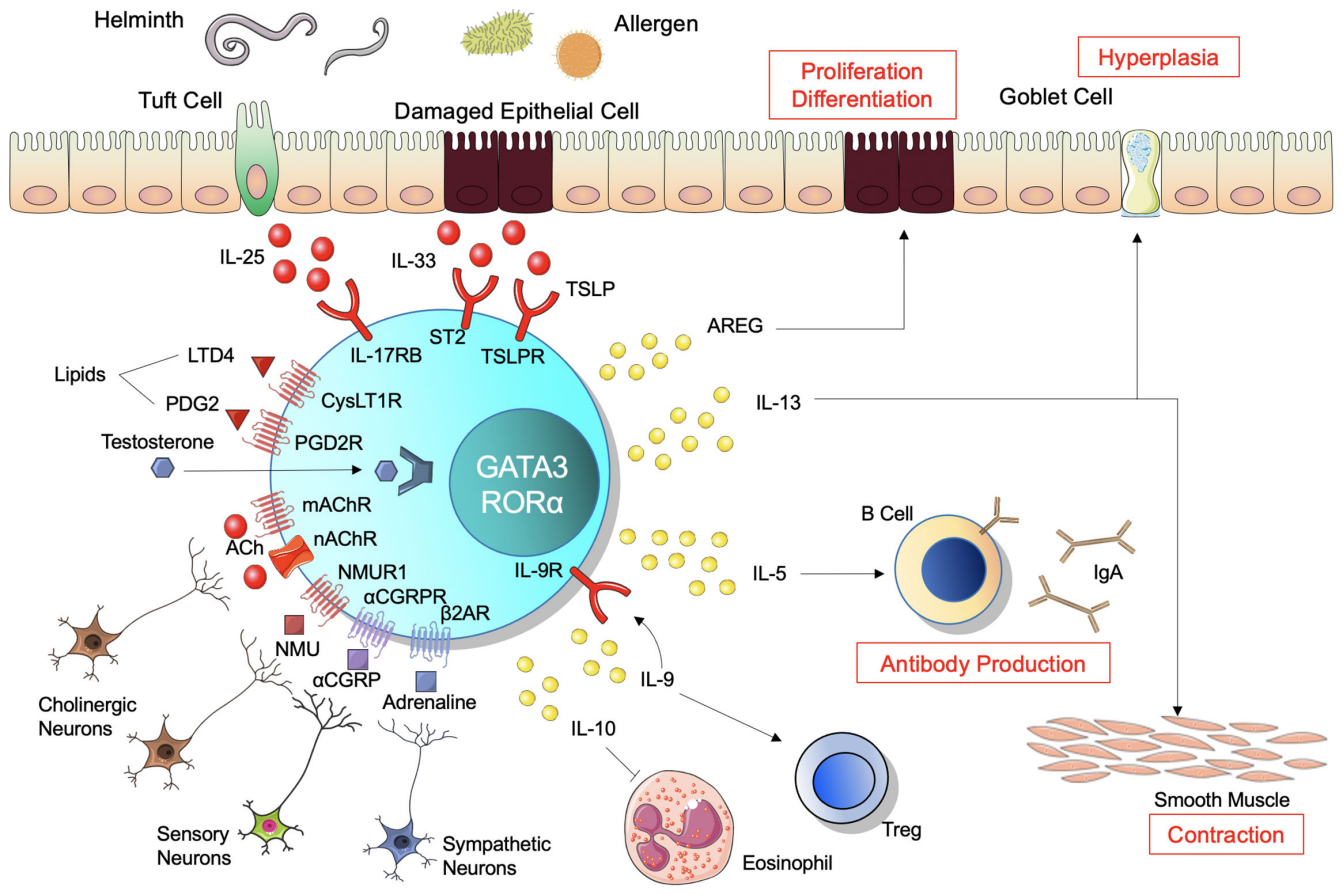
Regulation and function of ILC2. Alarmins, IL-25, IL-33, and thymic stromal lymphopoietin (TSLP), activate innate lymphoid cells 2 (ILC2) to expand and produce cytokines. Neural systems regulate ILC2s bidirectionally through neuropeptides and neurotransmitters. ILC2s require transcription factors GATA3 and RORα, and subsequently produce various cytokines. IL-13 induces hyperplasia of goblet cells and contraction of smooth muscle in the intestine, involved in clearance of antigens in the lumen. IL-5 regulates B-cell antibody production and enhances IgA production. Amphiregulin (AREG) promotes epithelial cell proliferation and differentiation. IL-9 and IL-10 contribute to resolution of inflammation, while IL-9 also promotes regulatory T-cell activation and IL-10 decreases eosinophil recruitment. ILC2 expresses IL-9 receptor and thus receives IL-9 autocrine feedback. Regarding ligands and receptors, red indicates activation, blue indicates inhibition, and purple indicates both functions.

ILC2s are localized in the lamina propria below the epithelial layer and are activated following epithelial damage by parasites and allergens in the mucosal tissues. This process is mediated by alarmins, such as IL-25, IL-33, and TSLP, which initially activate expansion and cytokine production in ILC2s when triggered by mucosal barrier damage (**Figure 1**). ILC2s responding to IL-33 produce the growth factor AREG, which binds to epithelium-expressed epidermal growth factor receptor (11). AREG has a critical role for epithelial cell proliferation and differentiation through the epidermal growth factor receptor pathway (21, 22). A recent study demonstrated that secretion of IL-33 was significantly accelerated in the colons of mice treated with dextran sulfate sodium (DSS) and injecting recombinant murine IL-33 improved epithelial damage, pro-inflammatory cytokine secretion, and loss of barrier function in DSS-induced colitic mice (23). Anti-colitic effect of IL-33 was observed in RAG2-/- or diphtheria toxin-treated DEREG mice where whole T cells or Tregs are depleted respectively (23). This suggests that ILC2 has significant roles in anti-colitic effect upon stimulation of IL-33 which is also known to enhance suppressive function of Foxp3+ regulatory T cells (Tregs) through its receptor ST2 (24) or stimulate CD103+ dendritic cells (DCs) to produce IL-2 and expand Tregs (25). In the small intestine, tuft cells, which exist in the epithelial layer of the intestinal tract and project microvilli into the lumen, produce IL-25 to sustain ILC2 homeostasis in the resting lamina propria (26). Together with high expression of IL-17RB (27), tuft cell-derived IL-25 activates ILC2s to produce IL-13, which affects epithelial crypt progenitors to promote differentiation of tuft and goblet cells, resulting in further activation of ILC2s in a positive feedback circuit of type 2 inflammation (26). This feed-forward pathway is constrained by CISH, a suppressor of cytokine signaling family member (28) and CISH-deficient ILC2s show excessive proliferation and cytokine production, resulting in increased tuft cells in the small intestinal (29). TSLP, which belongs to the IL-2 family with structural similarities to IL-7, is released from epithelial cells (30). TSLP incorporates the TSLP receptor (R) and the α-subunit of the IL-7R, and this ternary molecular complex activates multiple signaling pathways, such as the JAK1 and 2, STAT3 and 5, MAPK, PI3K, and NF-κB pathways (31–33). TSLP enhances the type 2 immune response, in particular the activation of ILC2s, resulting in increased type 2 cytokines IL-4, IL-5, and IL-13 (34–39). These effector cytokines are also regulated at the post-transcriptional level. Tristetraprolin, encoded by *Zfp36*, is an RNA-binding protein that destabilize mRNA. In the *Zfp36*-/- mice, ILC2 produces excessive *Il5* and *Il13* in the small intestine and other organs (40). Taken together, ILC2 in the intestine is regulated at the transcriptional and post-transcriptional level upon cytokine stimulation.

### Neuroimmunology: ILC2 Neuro-Immune Crosstalk

The gastrointestinal tract is one of the most innervated organs, particularly by enteric neurons and extrinsic sympathetic and parasympathetic nerves, such as the vagus nerve (41). This neuronal regulation shapes the levels of inflammation and homeostasis in the gut *via* controlling the epithelia, stroma, and immune cell compartments. The immune and neuronal systems interact bidirectionally, namely through neuropeptides and neurotransmitters that regulate immune cell functions, while inflammatory mediators from immune cells enhance neuronal activation. This “neuro-immune crosstalk” plays critical roles in tissue homeostasis (42–44). In addition to the intestinal macrophage (45), T-cell (46, 47), and ILC3 (48), ILC2s have been investigated for neuro-immune crosstalk from the early stages following identification (49, 50). ILC2s express receptors for neuropeptides and neurotransmitters, and are regulated through these receptors. Neuromedin U (NMU), a neuropeptide secreted from sensory cholinergic neurons, is detected in the intestine with high levels of expression and exerts biological activities through two G protein-coupled receptors: NMU receptor 1 (NMUR1) and 2 (NMUR2). NMUR1 is distributed in the peripheral tissues while NMUR2 is mainly observed in the central nervous system (51). Among the immune cells reported to express NMUR1 at a significant level, ILC2s predominantly express NMUR1 compared to other immune cell subsets, such as T cell, mast cell, and other groups of ILCs (52–56). NMU induces activation, proliferation, and type 2 cytokine secretion in ILC2s through NMUR1 (54–56). Although NMU regulation of ILC2s has been elucidated mainly in the field of allergic respiratory diseases, this relationship has also been revealed in the mouse gastrointestinal tract, indicating that NMU induces ILC2 activation, proliferation, and secretion of the type 2 cytokines IL-5, IL-9, and IL-13 (55). Calcitonin gene-related peptide (CGRP) is a later-identified neuropeptide that regulates ILC2s and is expressed and released by sensory neurons and ILC2s themselves (57–59). ILC2s express the receptor for α-CGRP in homeostatic and inflammatory conditions, and α-CGRP suppresses ILC2 proliferation by activating a cAMP response module, while promoting IL-5 expression (59). Single-cell RNA sequencing has revealed that expression of *Calca*, which encodes α-CGRP, is induced in intestinal killer-cell lectin like receptor G1 (KLRG1)-positive ILC2s in a food allergy model, but it is expressed in choline O-acetyltransferase (ChAT)^+^ sensory neurons in the steady state (59). These paradoxical functions of CGRP in terms of pro- and anti-inflammatory influence on immune responses may represent key roles for maintenance of epithelial cell homeostasis by adjusting immune responses to neuronal signals. In particular, IL-5 enhanced by α-CGRP promotes repair of epithelial cell damage, while α-CGRP prevents excessive type 2 inflammation by suppression of ILC2 proliferation (59). Of note, ChAT^+^ ILC2s are strongly induced by type 2 inflammatory conditions, such as helminth infection, *Alternaria* sensitization, and IL-25 and IL-33 treatment (38). In addition, ILC2s purified from the small intestine or cultured under IL-2, IL-7, and IL-33, express both muscarinic (*Chrm4* and *Chrm5*) and nicotinic (*Chrna2*, *Chrna5*, *Chrna9*, and *Chrna10*, *Chrnb1* and *Chrnb2*) acetylcholine (ACh) receptors. Therefore, ILC2s can respond to ACh to produce IL-5 and IL-13, and induce goblet cell hyperplasia, eosinophil accumulation, and helminth expulsion in the small intestine, which are partially abrogated in ILC-specific deletion of ChAT. Tuft cells also have the capacity to produce ACh and contribute to the regulation of ILC2s (26, 60). Studies suggest that the expression of CGRP and ChAT in ILC2s is similarly induced by type 2 inflammation and positive autocrine loops of ILC2-ACh or ILC2-CGRP potentially modify intestinal inflammation and homeostasis, and also raise an interesting question to identify the distinct roles of neuropeptides like CGRP and ACh released by ILC2s and sensory neurons. Although ILC2s respond to ACh both in the intestine and lung, ILC2s in the lung express the α7-nicotinic ACh receptor in contrast to intestinal ILC2s, suggesting tissue specificity of ACh receptor usage in ILC2s (61). Similar to the CGRP, another vasodilative neuropeptide, vasoactive intestinal polypeptide (VIP) is also involved in the regulation of ILC2s. Intestinal ILC2s express the VIP receptor and produce IL-5 when incubated with IL-7 and VIP (62). Reciprocally, IL-5 from ILC2s directly activates nociceptors, such as TRPV1 and TRPA1, on afferent Na_V_1.8^+^ neurons and upregulates the release of VIP, which induces ILC2s and T-cells to release more IL-5 and form a positive feedback loop of type 2 inflammation (63).

ILC2s are regulated by not only cholinergic neurons but also adrenergic neurons. The β2-adrenergic receptor, a catecholamine receptor expressed on ILC2s, recognizes noradrenaline released from sympathetic neurons and downregulates ILC2 function and type 2 inflammatory responses (64). Hence, both parasympathetic neurons releasing ACh and sympathetic neurons releasing noradrenaline affect suppression of ILC2-derived type 2 inflammation.

## ILC2 in Inflammatory Bowel Diseases

**Figure 2:**
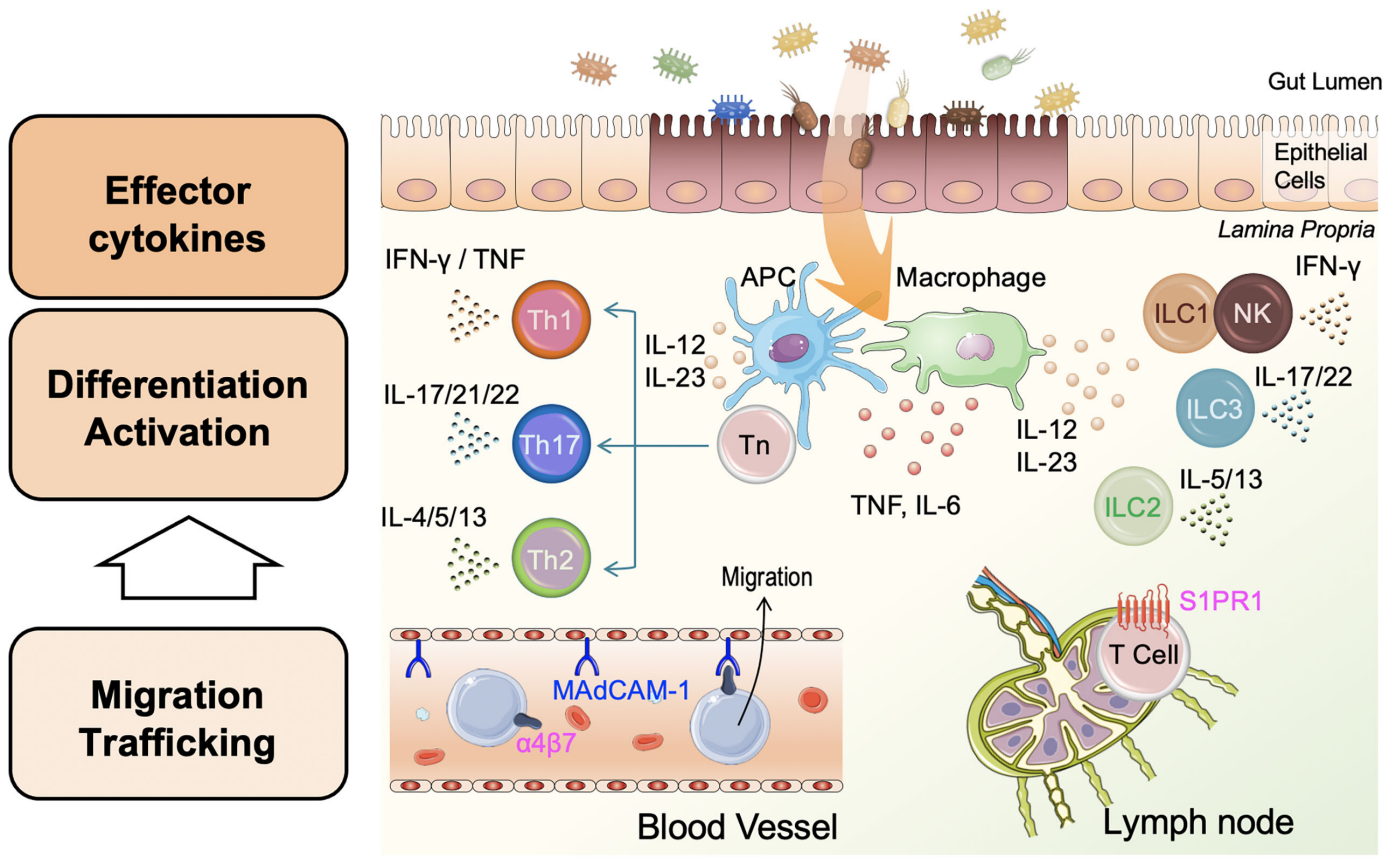
Pathophysiology of inflammatory bowel disease (IBD). Damaged epithelial cells enhance antigen presenting cell (APC) and macrophage uptake of antigens such as bacteria in the gut lumen, leading to APC/macrophage activation. APCs and macrophages produce pro-inflammatory cytokines, including tumor necrosis factor (TNF), IL-6, IL-12, and IL-23. Activated APCs present processed antigens to naïve helper T-cells (Tn) and promote the differentiation of Tn to effector T-cells, helper T-1 (Th1), Th2, and Th17 cells. Th1 and Th2 release type 1 cytokines (interferon [IFN]-γ and TNF), and type 2 cytokines (IL-4, IL-5, and IL-13), respectively. Independent of these, Th17 cells release IL-17, IL-21, and IL-22. Innate lymphoid cells 1 (ILC1s) and natural killer (NK) cells, ILC2s, and ILC3s are activated in a non-antigen-specific manner in tissues and produce cytokines corresponding to adaptive Th cell phenotypes, Th1, Th2, and Th17, respectively. Inflammation in the lamina propria is involved in the migration and trafficking of lymphoid cells from blood vessels and lymph nodes. Circulating lymphoid cells bearing integrin-α4β7 bind to the mucosal vascular epithelium through mucosal addressin-cell adhesion molecule 1 (MAdCAM-1) and migrate to the inflamed intestine. T-cells expressing shingosine-1-phosphase receptor 1 (S1PR1) in the lymph node are recruited to the site of inflammation by stimulation with S1P. Each cytokine or molecule has been targeted for IBD treatment.

IBD, mainly comprising UC and CD, is chronic inflammatory disease of the gastrointestinal tract, although the etiology of IBD remains unclear. To date, more than 200 IBD-associated genes have been identified and impinge on the pathways associated with cytokine signaling, bacterial recognition, and barrier function (65–67). Accumulation of many studies reveals that abnormal immune responses against microorganisms of the gut flora initiates chronic intestinal inflammation in genetically susceptible individuals (68). Furthermore, dysregulation of both innate and adaptive immune pathways contributes to the pathogenesis of IBD (69) (**Figure 2**). Detailed elucidation of the innate immune system, including ILCs, is required to gain new insights into the immunologic mechanisms of intestinal inflammation.

Compared with ILC1s and ILC3s, the role of ILC2s in IBD patients is less well understood (19). This might be attributed to the very low frequency of ILC2s in the entire human gastrointestinal tract compared with the relatively high abundance of ILC1s in the upper gastrointestinal tract and ILC3s in the ileum and colon (70). At the time of IBD diagnosis, the frequency of ILC1s is increased in patients with CD, and the frequency of NKp44+ ILC3s in inflamed tissue is decreased in both CD and UC patients (71), consistent with the previous literature showing that NKp44+ ILC3s produce IL-22 and IL-22-producing ILC3s are decreased in IBD (72–76). In contrast, the frequency of ILC2s is increased in patients with UC at diagnosis (71), while both ILC1s and ILC2s are increased in patients with IBD established for at least 1 year (71). Although reports of increased ILC2 frequency are traditionally present in CD but not UC (77), a recent study suggests the involvement of ILC2s with mucosal inflammation in both CD and UC. Impressively, ILC2s show plasticity towards an ILC1 cytokine profile with IL-12 stimulation, and some ILC2s in the mucosa of CD patients acquire capacity to produce IFN-γ in addition to IL-13, which could potentially contribute to intestinal inflammation (78). IL-12 is expressed and actively released in CD intestinal tissues (79), and is the therapeutic target of ustekinumab, which is used to treat CD and UC (80, 81). Notably, in IBD patients receiving vedolizumab, a monoclonal antibody that targets integrin α4-β7 and blocks gut-homing of activated immune cells, the frequencies of ILCs in peripheral blood remained unchanged, suggesting that distribution of ILCs is due to local proliferation or plasticity rather than recruitment of ILCs to the intestine (71). Therefore, the finding of increased frequency of ILC2s secreting IFN-γ may indicate that the plasticity of ILCs depends on the local mucosal microenvironment.

IL-33 expression is enhanced in the inflamed mucosa of IBD patients (24, 82) and experimental models of colitis (83), and has been previously shown to play both protective and detrimental roles in colitis, based on different models of colitis and analyses of cell types. Genetic ablation of ST2, a receptor of IL-33, resulted in amelioration of colitis induced by DSS or trinitrobenzene sulfonic acid (84). In addition, ILC2s expand and produce more Th2 cytokines during DSS-induced colitis, which is repressed in the steady state by E-cadherin on colonic epithelial cells and KLRG1 on ILC2s (85). Conversely, treatment with IL-33 or transfer of ILC2s improve intestinal mucosal damage through the AREG pathway in the DSS-induced colitis model (11). A recent study has reported that the intracellular pattern recognition receptor NOD2 drives early IL-33-dependent expansion of ILC2s during CD ileitis, based on CD patient samples and an established murine model of CD-like ileitis, the SAMP1/YitFc mouse strain (86, 87). In addition to alarmins, CC chemokine ligand 1 (CCL1) exerts unique roles on ILC2s in the intestine. ILC2s express high levels of the Th2-type chemokine receptor, C-C motif chemokine receptor 8 (CCR8), both in mouse intestine and human peripheral blood. In addition, the expression of CCR8 and its ligand CCL1 is upregulated in patients with UC and in the DSS-induced colitis model (88). In a helminth infection model, mice lacking CCR8 exhibit reduced type 2 cytokines IL-5, IL-13, and IL-9, and greater worm burden in the small intestine (89). This is not attributed to aberrant migration but to impaired proliferation and cytokine production in ILC2s in the lung and intestine, although CCL1/CCR8 signaling contributes to mediating monocyte and lymphocyte chemoattraction and is implicated in vascular regulatory T-cell recruitment and function (90). Of note, ILC2s are the major producers of CCL1, which forms a paracrine CCL1/CCR8 feed-forward loop during helminth clearance (89). Unlike parasite infection, the major source of CCL1 during DSS-induced colitis is macrophages rather than ILC2s, but CCL1/CCR8 signaling similarly protects hosts from both parasite infection and acute intestinal damage in a DSS colitis model (88). In addition, mice lacking CCR8 exhibit comparable numbers of ILC2 and tissue-repairing cytokines, IL-10 and AREG, but reduced numbers of intestinal IFN-γ-producing ILCs (88). However, these IFN-γ-producing ILCs may also have dual roles in colitis as discussed above. Further studies are needed to disentangle the complex results of previous reports regarding the roles of ILC2s in colitis and clinical trials targeting Th2 cytokines, and to further enhance our comprehension of the contribution of ILC2s to immune mechanisms in IBD.

### ILC2 Contribution to Intestinal Fibrosis

Inflammation and impaired tissue repair induce accumulation of myofibroblasts, which produce extracellular matrix components, resulting in organ fibrosis (91, 92). In the intestine, fibrosis can lead to stenosis or perforation. Th2 cells produce type 2 cytokines, IL-4, IL-5, and IL-13, generating various pathological changes, such as infiltration of eosinophils, increased mucus production, and fibrosis (93). Recent studies have revealed that not only Th2 cells but also ILC2s producing type 2 cytokines in an antigen non-specific manner play an important role in immune-mediated fibrosis and modulation of tissue remodeling, causing dysfunction in various organs. Regarding the lung, expression of IL-25 and the ILC2 population increase in the lungs of idiopathic pulmonary fibrosis patients (94). Other alarmin cytokines, IL-33 and TSLP, are elevated in idiopathic pulmonary fibrosis, cystic fibrosis, and steroid-resistant asthma sufferers (95–98). These studies suggest that alarmin cytokines have critical roles in lung fibrosis and remodeling. In patients suffering from liver fibrosis of various etiologies, such as virus infection, alcoholic liver disease, non-alcoholic steatohepatitis, autoimmune hepatitis, and primary cholangitis (99), numbers of liver-resident ILC2s are activated and expanded followed by expression of IL-33 (100). A recent study has revealed the contribution of ILC2s in skin fibrosis within systemic sclerosis (101). Following activation by IL-33, ILC2s express the growth factor AREG and participate in epithelial barrier function and tissue repair in the intestine (11).

IL-13 produced by ILC2s is involved in expression of the tumor necrosis factor family cytokine TL1A, overexpression of which brings about intestinal fibrosis (102). Constitutive expression of TL1A in lymphoid and myeloid cells leads to spontaneous inflammation and fibrosis in the small intestine and colon (103, 104). TL1A is a ligand for death receptor 3 and enhances secretion of pro-inflammatory cytokines through multiple cell lineages (105). ILC2s highly express death receptor 3 and overexpression of TL1A activates ICL2 expansion, independent of IL-25 or IL-33 (106). Notably inhibition of TL1A function by either anti-TL1A neutralizing antibody or deletion of death receptor 3 reduces numbers of intestinal fibroblasts and myofibroblasts in murine DSS colitis, the model of human IBD (107). Deficiency of another tumor necrosis factor family cytokine, LIGHT, in mice exacerbates DSS colitis compared with controls and accumulates ILCs, suggesting LIGHT plays roles in regulating inflammation in the colon (108). Signaling through LIGHT receptor, lymphotoxin β receptor, in epithelial cells and dendritic cells protects against mucosal damage by inducing IL-22 from ILC3s (109). Although *Tnfsf14*, the gene encoding LIGHT, is highly expressed in not only ILC3s but also ILC2s (108, 110), the role of the LIGHT-lymphotoxin β receptor interaction in ILC2s has not yet been revealed and further research is needed.

Blocking IL-13 production from ILC2s by IL-25 neutralization enhances IL-22 production from ILC3s, which repair epithelial damage (111, 112). In murine models, IL-13 is associated with chronic gut inflammation caused by trinitrobenzene sulfonic acid (113) and triggers transforming growth factor β 1-dependent fibrosis (114). Notably, IL-13 was identified as the key effector cytokine in UC by affecting epithelial apoptosis, tight junctions, and restitution velocity (115) and a promotor of collagen accumulation in CD by inhibiting fibroblast matrix metalloproteinase synthesis, resulting in fibrosis of intestine tissue (116). These studies indicate that blockade of IL-13 improves inflammation and subsequent fibrosis in IBD patients. However, clinical trials evaluating tralokinumab, an anti-IL-13 neutralizing antibody for moderate-to-severe UC (117), and anrukinzumab, an anti-IL-13 monoclonal antibody for mild-to-moderate UC (118), could not demonstrate statistically significant therapeutic effects compared with placebo controls. Although the effect of inhibiting IL-13 for IBD patients remains controversial, a recent study that found a high frequency of autoantibodies against integrin αvβ6 in UC patients suggests the possible contribution of type 2 immune responses in the pathogenesis in IBD (119).

## The Roles of ILC2 for infection and Allergy

As mentioned above, the exposure to pathogens such as parasites and allergens triggers ILC2 activation in mucosal tissue. Parasites and allergens contain catalytic enzymes that digest the mucosal barrier and provoke massive epithelial cell death, leading to release of IL-33, which rapidly activates ILC2s in the lung (120) and colon (121, 122). Since IL-33 rescues RAG2-/-, but not RAG2-/- γc-/-, mice from *Clostridioides difficile* (121) and amebic (122) infection, IL-33-ILC2 exerts host protection from these intestinal infections. In the nucleus of epithelial cells, endogenous IL-33 is highly expressed upon tissue inflammation (123). Additionally, lipid chemical mediators play critical roles in ILC2 activation (124, 125). ILC2s in the lung from wild-type, RAG2-/-, and STAT6-/- mice express cysteinyl leukotriene receptor 1 (CYSLTR1), and are induced to produce IL-4, IL-5, and IL-13 followed by stimulation of leukotriene D4 (124). Similar to the lung ILC2, small intestine ILC2 expresses CYSLTR1 and CYSLTR2, and produces IL-13 upon stimulation of leukotriene C4 and D4 (126). In small intestine, tuft cells become the essential source of cysteinyl leukotriene and activate ILC2s in cooperation with IL-25 following helminth infection (126). An *in vitro* study of ILC2s isolated from human skin showed that prostaglandin D2 induces ILC2 migration, production of type 2 cytokines and other pro-inflammatory cytokines, and upregulation of the expression of IL-33R and IL-25R (125). A subsequent study demonstrated that testosterone attenuates ILC2 function, and this result may explain the sex difference in prevalence of allergic disease (127). However, these ILC2 regulatory mechanisms have not yet been demonstrated in the intestinal tract.

Activated ILC2s exert inflammatory responses mainly *via* type 2 cytokines. IL-5, IL-6, and IL-13 are ILC2-derived cytokines that were identified when ILC2s were first discovered (13). IL-5 regulates B-cell antibody production and enhances IgA production from B-cells, while IL-5 and IL-13 are implicated in allergic inflammation and protection against helminth infection (13, 128). The recent study demonstrates that ILC2s predominate in the stomach, are induced by commensal bacteria, and protect against *H. pylori* infection through B cell activation and IgA production (129). IL-13 promotes intestinal smooth muscle contractility for exclusion of enteric nematode parasites and is required for expression of STAT6 (130). IL-13 derived from ILC2s induces hyperplasia of goblet cells, the columnar epithelial cell that lines gastrointestinal mucous membrane and contains abundant mucin, and participates in clearance of luminal antigens (131, 132). While activated ILC2s produce large amounts of IL-5 and IL-13, the level of IL-4 is generally low except in specific inflammatory conditions or disease models (14, 133, 134). IL-4 released from ILC2s promotes food allergy by blocking allergen-specific regulatory T-cells (135), and is required for type 2 helper T-cell (Th2) differentiation following helminth infection (136). Alternatively, ILC2s can also respond to IL-4 derived from eosinophils or basophils and accelerate proliferation and activation of ILC2s themselves. This feed-forward loop contributes to amplification of type 2 inflammation (137, 138). ILC2s also produce IL-9 following activation by IL-33, but not IL-25 (139). IL-9 derived from ILC2s promotes regulatory T-cell activation and effectively induces resolution of inflammation (140). Moreover, ILC2 simultaneously expresses IL-9R during helminth infection, suggesting an autocrine feedback of ILC2-derived IL-9 (139). Conversely, a molecularly distinct subset of ILC2s produce IL-10 following IL-2 activation and subsequently decrease eosinophil recruitment, suggesting downregulation of inflammation (12). Consequently, ILC2s interact with other immune cells through various cytokine crosstalk pathways and form amplification loops of type 2 immune responses with Th2 cells, eosinophils, and basophils.

## Heterogeneity and Plasticity of ILC2

Recent studies have shown the heterogeneity of ILC2 subsets between tissues and implicated environmental factors in this variability. In the lung, the existence of two different ILC2 subsets—inflammatory ILC2 (iILC2) and natural ILC2 (nILC2)—have been identified, and these have different phenotypes, such as ST2 (a heterodimer of IL-33R), Thy1, KLRG1, and IL-17RB (10). iILC2 cells express more IL-25R and develop into nILC2-like cells, producing IL-5 and IL-13 after stimulation with IL-33 during worm infection. Moreover, iILC2 migrate from the intestinal lamina propria to other organs, including lung and liver, dependent on chemotaxis mediated by sphingosine 1-phosphate after injection of IL-25 or helminth infection (141). Although ILC2s are largely tissue-resident (142, 143), the ability of ILC2s to migrate suggests that ILC2s complement adaptive immunity by protecting both local and distant tissue against infection.

In the small intestine, IL-33 promotes the generation of iILC2s by induction of tryptophan hydroxylase 1, deletion of which results in increased susceptibility to helminth infection (144). However, in the colon, ILC2s express ST2. Following administration of IL-33, these cells proliferate and demonstrate high expression of IL-5 and IL-13, with lower expression of IL-17 (145). Compared with other organs such as lung or skin, ILC2s in the small intestine express higher levels of IL-17RB, which forms the IL-25R together with IL-17RA (146, 147). Enriched IL-17RB in intestinal ILC2s suggests that IL-25 derived from tuft cells promotes efficient activation of ILC2s and defense against infection with helminths or other pathogens (26, 27, 148). Although TSLP is primarily expressed in skin keratinocytes, lung, and gut epithelial cells (31), the function of TSLP in the gut has not yet been clearly identified in contrast to its role in allergy and infection in the lung and skin (149).

The first cell population-specific RNA sequence study to characterize murine ILC subsets in the lamina propria of the small intestine identified the expression of genes associated with lipid metabolism, such as *Dgat2*, *Pparg*, and *Lpcat2*, and a gene associated with enteric neuron communication, *Bmp2* (147). In another single-cell sequencing study, graded expression of GATA3 characterized four different groups of intestinal ILC2s (150). ILC2s, which express high levels of marker genes, such as *Klrg1*, *Klf4*, *Ly6a*, and *Il2ra*, uniquely expressed high levels of *Il5*, *Csf2*, and *Areg* (150). As described above, single-cell RNA sequencing assists to determine the heterogeneity of ILC2s, particularly in the field of neuroimmunology. In studies of the lung, ILC2s in *Nippostrongylus brasiliensis*-infected mice are clustered into four subsets: resting nILC2s, *Il5*-high nILC2s, *Il13*-high nILC2s, and iILC2s (57). The expression of α-CGRP receptor is enriched within an *Il5*-high subpopulation of ILC2s and α-CGRP promotes IL-5 production only at early time point stimulations (57). Intestinal ILC2s express the components of the α-CGRP receptor at steady state, while α-CGRP suppresses the proliferation of ILC2s, but increases IL-5 levels during the early inflammatory phase (59). Furthermore, ILC2s of the small intestine express abundant NMUR1 gene, while adaptive immune cells, ILC1s, and ILC3s do not (55).

*In vitro* studies demonstrate the plasticity of human ILC2s, which switch phenotype between subsets such as ILC1s and ILC3s. IL-12 promotes the conversion of ILC2s into ILC1-like cells, characterized by expression of T-bet and production of IFN-γ (78, 138, 151). Conversely, IL-4 derived from eosinophils promotes ILC2 maintenance and proliferation by preventing IL-12-mediated ILC2 differentiation into the ILC1 phenotype (138). Furthermore, the ILC2 subpopulation that expresses c-Kit can convert into ILC3-like cells, producing IL-17 in response to IL-1β and IL-23 (152). Removal of the aryl hydrogen receptor, a transcription factor for ILC3, activates intestinal ILC2s, whereas increased aryl hydrogen receptor expression suppresses ILC2 function and enhances ILC3 function (153). As ILC3-to-ILC1 conversion has been reported (19, 154), ILC2s also demonstrate plasticity, resulting in ILC2 heterogeneity in the inflammatory gut.

## Conclusion

ILC2s play important roles not only for protection against infection but also for promotion of chronic inflammation and tissue fibrosis. A variety of cytokines and cellular interactions with other immune cells and neuronal systems are involved in the homeostasis of ILC2s, suggesting complexity of ILC2 regulation. Recent studies revealed the potential of intestinal ILC2s, such as migration to other organs and plasticity of conversion to ILC1s or ILC3s. In particular, single-cell analysis can help our understanding of heterogeneity of ILC2s potentially attributed to pathological mechanisms and aid in discovery of therapeutic targets for chronic inflammation, including IBD. Understanding uniqueness and commonness of ILC2 between mice and humans, between the gut and other organs, and between health and disease may help answer important questions in gut biology: What role does ILC2 play in the contexts such as IBD, infectious disease, colorectal cancer, food allergy and intestinal fibrosis? What is the unique role of each ILC2 subset in the clinical settings? How are these ILC2 subsets dynamically regulated during the course of intestinal disease? How does each ILC2 subset interact with the other ILC2 subsets and other types of immune cells? What factors contribute to diversification of ILC2 subsets? Can these ILC2 subsets be targeted to develop effective therapeutic strategies for human intestinal diseases? Further research on ILC2s in different environments at different phases of intestinal inflammation will provide a clearer view on the roles of ILC2 during colitis, tissue regeneration, fibrosis, and cancer.

## Author Contributions

SS wrote the first draft of the manuscript and figures. JT wrote sections of the manuscript and drafted the figures. YM conceived, supervised, revised the text and figures. TK and TT supervised the study. All authors contributed to the article and approved the submitted version.

## Funding

This study was funded by the Japan Society for the Promotion of Science (JSPS) KAKENHI (B) 20H03666 to YM, and (A) 20H00536 to TK; JSPS Grant-in-Aid for Transformative Research Areas(B): 21H05123 to YM; Advanced Research and Development Programs for Medical Innovation (AMED-CREST: 16gm1010003h0001 and 21gm1510002h0001to TK, and 20gm1210001h0002 to YM; the Practical Research Project for Rare/Intractable Disease: 21ek0109556h0001 to YM); the Japan Foundation for Applied Enzymology; and Keio University Medical Fund. The authors declared no conflicts of interest.

## Conflict of Interest

The authors declare that the research was conducted in the absence of any commercial or financial relationships that could be construed as a potential conflict of interest.

## Publisher’s Note

All claims expressed in this article are solely those of the authors and do not necessarily represent those of their affiliated organizations, or those of the publisher, the editors and the reviewers. Any product that may be evaluated in this article, or claim that may be made by its manufacturer, is not guaranteed or endorsed by the publisher.
